# Comparisons of ELISA and Western blot assays for detection of autophagy flux

**DOI:** 10.1016/j.dib.2017.06.045

**Published:** 2017-07-04

**Authors:** Sung-hee Oh, Yong-bok Choi, June-hyun Kim, Conrad C. Weihl, Jeong-sun Ju

**Affiliations:** a Department of Exercise Science, Research Institute of Sports Science, the University of Suwon, 17 Wauan-gil, Bongdam-eup, Hwaseong-si, Gyeonggi-do 18322, South Korea; b Department of Biotechnology and Bioscience, School of Bioindustry, the University of Suwon, 17 Wauan-gil, Bongdam-eup, Hwaseong-si, Gyeonggi-do 18322, South Korea; c Department of Neurology, Washington University School of Medicine, PO Box 8111, 660 South Euclid Avenue, St Louis, MO 63110, USA

**Keywords:** Autophagy, Mitophagy, ELISA, Western blot, Skeletal muscle

## Abstract

We analyzed autophagy/mitophagy flux *in vitro* (C2C12 myotubes) and *in vivo* (mouse skeletal muscle) following the treatments of autophagy inducers (starvation, rapamycin) and a mitophagy inducer (carbonyl cyanide m-chlorophenylhydrazone, CCCP) using two immunodetection methods, ELISA and Western blotting, and compared their working range, accuracy, and reliability. The ELISAs showed a broader working range than that of the LC3 Western blots (Table 1). Table 2 showed that data value distribution was tighter and the average standard error from the ELISA was much smaller than those of the Western blot, directly relating to the accuracy of the assay. Test-retest reliability analysis showed good reliability for three individual ELISAs (interclass correlation, ≥ 0.7), but poor reliability for three individual Western blots (interclass correlation, ≤ 0.4) (Table 3).

**Specifications Table**

**Table:** 

Subject area	Biochemistry
More specific subject area	Assay development and method
Type of data	Tables
How data was acquired	enzyme-linked immunosorbent assay (ELISA), Western blotting
Data format	Descriptive data: mean±SD, analyzed
Experimental factors	C2C12 myotubes and male wild-type C57BL/6 mouse skeletal muscle
Experimental features	*in vitro* and *in vivo* autophagy/mitophagy flux were measured using two immunodetection techniques
Data source location	Hwaseong, South Korea
Data accessibility	Data are available with this article

**Value of the data**

• The presented data indicated that the ELISA had smaller data distribution and was more repeatable to measure autophagy/mitophagy flux, compared with Western blot data.
• These data could be helpful for many autophagy researchers to obtain more accurate and reproducible data using this ELISA technique.
• These data could also be beneficial for researchers in other areas to adapt the ELISA-based assay strategy from the Western blot.

## 1 Data

We compared the ELISA with Western blot data from C2C12 myotubes and male wild-type C57BL/6 mouse skeletal muscle tissue in terms of the working range, accuracy, and reliability for autophagy/mitophagy flux measurements. The ELISAs could surpass the Western blot in all criteria for quantification of autophagy flux. The ELISAs showed a broader dynamic range than the Western blot (5.3 *versus* 1.4, the ratio of the highest O.D. value to the lowest) (Table 1). The values of standard error from the ELISA were much smaller than those of the Western blot (*P*<0.05, Table 2), which implies the accuracy of the assays. When each of the three individual assays was compared, the ELISA was more reliable than the Western blot (interclass correlation, ≥ 0.7 *versus* ≤ 0.4) (Table 3).

**Table 1:** The working range of the ELISA and the LC3 Western blot.

**ELISA**	**Blanks**	**Lowest O.D.**	**Highest O.D.**
Average values	0.03±0.002	0.183±0.028	0.961±0.031
Ratio to blanks		6	32
Dynamic range			5.3^1^
**Western blot**	**Background**	**Lowest density**	**Highest density**
Average values	0.071±0.005	0.301±0.093	0.407±0.052
Ratio to background		4.2	5.7
Dynamic range			1.4^1^

Values are means±SD; *n*=18/group.

1 The ratio of the highest O.D. value to the lowest.

**Table 2:** Comparisons of average standard errors of data from C2C12 cells and TA muscles treated with starvation, rapamycin, and CCCP.

Average Standard Error			
**C2C12 Cells**	**Starvation**	**Rapamycin**	**CCCP**
Western blot	0.180±0.082	0.240±0.088	0.172±0.037
ELISA	0.07±0.009	0.161±0.013	0.018±0.005
**Fold difference**	2.6^*^	1.5^*^	9.6^*^
**TA muscles**	**Starvation**	**Rapamycin**	**CCCP**
Western blot	0.778±0.105	0.301±0.093	0.407±0.052
ELISA	0.041±0.014	0.042±0.006	0.032±0.003
**Fold difference**	19.0^*^	7.2^*^	12.7^*^

Values are means±SD; *n*=18/group. Difference was evaluated using a paired *t*-test at *P*<0.05.*Fold change compared to control groups.

**Table 3:** Test-retest reliability for three inter-assays.

**3 ELISAs**	**ICC**	**95% CI**

Rapamycin	0.889	0.53, 0.98
CCCP	0.778	0.06, 0.966
**3 Western blots**	**ICC**	**95% CI**
Starvation	0.3804	−1.63, 0.91
Rapamycin	0.234	−2.25, 0.88
CCCP	0.077	−2.911, 0.86

ICC: intraclass correlation, CI: confidence interval.

## 2 Experimental design, materials and methods

We performed the autophagic flux assays using *in vitro* and *in vivo* LC3-II turnover measured by Western blot in the presence and absence of an autophagy inhibitor as previously described [1], [2] and the ELISA using LC3 antibodies following subcellular fractionation of mouse skeletal muscle cells and tissue as described in a recent paper [3].

### 2.1 Measurement of autophagic flux in cultured cells

C2C12 myotubes grown on 10-cm dishes were incubated in amino acid and serum-free starvation buffer or treated with 10 μg/mL rapamycin or 25 μg/mL CCCP with and without 200 nM BafilomycinA1 for 8 h.

### 2.2 Measurement of autophagy flux in animals

Ten week old male wild-type C57BL/6 mice were divided into four groups: fed and starvation, or vehicle, rapamycin, and CCCP. Starvation was performed by removing food for 48 h. 10 mg/kg/day rapamycin, 4 mg/kg/day CCCP or vehicle was *i.p.* injected to mice daily for two days. Mice were also treated with and without 0.4 mg/kg/day colchicine administration for 48 h. Control mice received an equal volume of *i.p.* saline.

### 2.3 Membrane/cytosol fractionation

Subcellular fractionations from cultured cells or tissue were performed as previously described [4].

### 2.4 Mitochondrial/cytosol fractionation

Mitochondrial fractionations from cultured cells or tissue were performed as previously described [5].

### 2.5 Sandwich ELISA assay

Following subcellular fractionation of mouse skeletal muscle cells and tissue, cytosolic, membrane, and mitochondrial fractions were analyzed through a sandwich ELISA using two LC3 antibodies, LC3 capture and HRP-conjugated LC3 detection antibodies as previously described [3].

### 2.6 Immunoblot analysis

LC3 protein turnover and other protein levels were measured using immunoblot techniques as previously described [3].

### 2.7 Statistical analysis

Data are presented as means±SD and were evaluated by a paired t-test or reliability analysis at *p*<0.05.

## Funding

This work was supported by the National Research Foundation of Korea Grant funded by the Korean Government (NRF-2013-332-2013S1A5A8021905).
