# Supplementary material for: Comparisons of ELISA and Western blot assays for detection of autophagy flux
Source: Data Brief. 2017 Jul 4;13:696–9. doi: 10.1016/j.dib.2017.06.045 (PMC5506881; doi:10.1016/j.dib.2017.06.045)
Supplement: Supplementary file 1 — Supplementary material [file mmc1.docx]

*Data article*

**Title**: Data Comparisons of ELISA and Western blot assays for detection of autophagy flux

**Authors:** Sung-hee Oh^a^, Yong-bok Choi^b^, June-hyun, Kim^b^, Conrad C. Weihl^c^, Jeong-sun Ju^a,*^

**Conflict of interest:** The author(s) declare that they have no competing interests.
